# Evaluation of the Activity of Lamivudine and Zidovudine against Ebola Virus

**DOI:** 10.1371/journal.pone.0166318

**Published:** 2016-11-30

**Authors:** Yu Cong, Julie Dyall, Brit J. Hart, Lisa Evans DeWald, Joshua C. Johnson, Elena Postnikova, Huanying Zhou, Robin Gross, Oscar Rojas, Isis Alexander, Nicole Josleyn, Tengfei Zhang, Julia Michelotti, Krisztina Janosko, Pamela J. Glass, Mike Flint, Laura K. McMullan, Christina F. Spiropoulou, Tim Mierzwa, Rajarshi Guha, Paul Shinn, Sam Michael, Carleen Klumpp-Thomas, Crystal McKnight, Craig Thomas, Ann E. Eakin, Kathleen G. O’Loughlin, Carol E. Green, Paul Catz, Jon C. Mirsalis, Anna N. Honko, Gene G. Olinger, Richard S. Bennett, Michael R. Holbrook, Lisa E. Hensley, Peter B. Jahrling

**Affiliations:** 1 Integrated Research Facility, Division of Clinical Research, National Institute of Allergy and Infectious Diseases, National Institutes of Health, Frederick, Maryland, United States of America; 2 United States Army Medical Research Institute of Infectious Diseases, Frederick, Maryland, United States of America; 3 Viral Special Pathogens Branch, Division of High-Consequence Pathogens and Pathology, Centers for Disease Control and Prevention, Atlanta, Georgia, United States of America; 4 The National Center for Advancing Translational Sciences, National Institutes of Health, Bethesda, Maryland, United States of America; 5 Office of Biodefense, Research Resources & Translational Research, Division of Microbiology & Infectious Diseases, National Institute of Allergy and Infectious Diseases, National Institutes of Health, Rockville, Maryland, United States of America; 6 SRI International, Menlo Park, California, United States of America; 7 Emerging Viral Pathogens Section, National Institute of Allergy and Infectious Diseases, National Institutes of Health, Frederick, Maryland, United States of America; Helmholtz Zentrum Munchen Deutsches Forschungszentrum fur Umwelt und Gesundheit, GERMANY

## Abstract

In the fall of 2014, an international news agency reported that patients suffering from Ebola virus disease (EVD) in Liberia were treated successfully with lamivudine, an antiviral drug used to treat human immunodeficiency virus-1 and hepatitis B virus infections. According to the report, 13 out of 15 patients treated with lamivudine survived and were declared free from Ebola virus disease. In this study, the anti-Ebola virus (EBOV) activity of lamivudine and another antiretroviral, zidovudine, were evaluated in a diverse set of cell lines against two variants of wild-type EBOV. Variable assay parameters were assessed to include different multiplicities of infection, lengths of inoculation times, and durations of dosing. At a multiplicity of infection of 1, lamivudine and zidovudine had no effect on EBOV propagation in Vero E6, Hep G2, or HeLa cells, or in primary human monocyte-derived macrophages. At a multiplicity of infection of 0.1, zidovudine demonstrated limited anti-EBOV activity in Huh 7 cells. Under certain conditions, lamivudine had low anti-EBOV activity at the maximum concentration tested (320 μM). However, lamivudine never achieved greater than 30% viral inhibition, and the activity was not consistently reproducible. Combination of lamivudine and zidovudine showed no synergistic antiviral activity. Independently, a set of *in vitro* experiments testing lamivudine and zidovudine for antiviral activity against an Ebola-enhanced green fluorescent protein reporter virus was performed at the Centers for Disease Control and Prevention. No antiviral activity was observed for either compound. A study evaluating the efficacy of lamivudine in a guinea pig model of EVD found no survival benefit. This lack of benefit was observed despite plasma lamivudine concentrations in guinea pig of about 4 μg/ml obtained in a separately conducted pharmacokinetics study. These studies found no evidence to support the therapeutic use of lamivudine for the treatment of EVD.

## Introduction

Ebola virus (EBOV) was first isolated in 1976 in the Democratic Republic of the Congo (formerly Zaire) and subsequently identified as the causative agent in outbreaks of severe hemorrhagic fever in several countries in central Africa [1–3]. The recent EVD epidemic in Western Africa began in Guinea in December 2013, and as of June 10, 2016, a total number of 28,616 cases were reported worldwide with 11,310 deaths [4]. Clinical manifestations of EVD include acute febrile disease with flu-like symptoms, followed by diarrhea, vomiting, and impaired liver and kidney function, with potential development of severe hemorrhage and multi-organ failure [3, 5–7].

Currently, no licensed vaccines or drugs are available for prevention or treatment of EVD, and standard care consists of supportive therapy, including rehydration with oral or intravenous fluids and treatment of specific symptoms. Many researchers are evaluating a wide array of experimental drugs that specifically inhibit EBOV, but none of these drugs are clinically approved by any regulatory authority. Clinical trials and the approval process for novel drugs can take up to 15–20 years [8]. As an alternative, compounds with anti-EBOV activity that are already approved for other indications have been considered for repurposing [9, 10].

In September 2014, Dr. Gorbee Logan from Liberia reported successful treatment of 13 out of 15 patients with suspected EVD with lamivudine, an FDA-approved drug for treating HIV-1 infections [11]. However, details are limited regarding the treatment dose and regimen the patients received. The role, if any, that treatment with lamivudine had in the recovery of the patients is unclear. In addition, clinical confirmation of EVD in these cases remains to be verified.

Lamivudine is a reverse transcriptase inhibitor of human immunodeficiency virus-1 (HIV-1) and hepatitis B virus (HBV) and is a weak inhibitor of mammalian α**-**, β**-**, and γ**-**DNA polymerases [12, 13]. As a nucleoside analog, lamivudine inhibits retroviral reverse transcriptase by incorporating the active triphosphate form of lamivudine into viral DNA leading to chain termination [14]. Theoretically, lamivudine should not have an effect on replication of viruses that do not utilize reverse transcriptase for virus replication. We previously reported lamivudine had no antiviral activity against EBOV in Vero E6, Hep G2, or monocyte-derived macrophages (MDMs) infected with the 1995 EBOV Kikwit variant [15]. Given the continued interest of using lamivudine to treat EVD and the results of a recent article showing *in vitro* antiviral activity against EBOV infection [16], we further evaluated the efficacy of lamivudine and a second HIV-1 drug, zidovudine, in a broad range of infected cell lines. Tested cell lines included Vero E6, Huh 7, HeLa, Hep G2, and 293T cells, and primary MDMs, using varying multiplicities of infections (MOIs), drugs from different sources, and different time courses of drug treatment and assay endpoints. The potential for synergistic anti-EBOV activity of these drugs *in vitro* was also evaluated. To examine further the potential of lamivudine as a prospective treatment for EVD, an *in vivo* pilot study was conducted evaluating efficacy of the drug in a guinea pig model of EVD. These studies found no evidence to support the therapeutic use of lamivudine for the treatment of EVD.

## Material and Methods

### Drugs and Cells

Toremifene citrate (CAS 89778-27-8; T7204-5MG) and lamivudine (CAS 134678-17-4; L1295-10MG) were purchased from Sigma-Aldrich (St. Louis, MO). Lamivudine oral solution was purchased from ViiV Healthcare (Research Triangle Park, NC) or from Haller’s Pharmacy and Medical Supply (Fremont, CA), lamivudine powder was purchased from Sigma Aldrich. Zidovudine (zidovudine syrup) was obtained from Aurobindo Pharma, Ltd. (Dayton, OH). Lamivudine, zidovudine, and imatinib mesylate used in experiments conducted at the Centers for Disease Prevention and Control (CDC) were obtained from the United States Pharmacopeia Convention (Rockville, MD).

Vero C1008 (E6) kidney cells (African green monkey, working cell bank NR-596) were obtained through BEI Resources (National Institute of Allergy and Infectious diseases [NIAID], National Institutes of Health [NIH], Manassas, VA). Vero E6 (ATCC 1586), Hep G2 (ATCC HB-8065), HeLa (ATCC CCL-2), and 293T/17 (ATCC CRL-11268) cells were obtained from the American Type Culture Collection (Manassas, VA). Huh 7 (human hepatocellular carcinoma) cells were obtained from Dr. Hideki Ebihara (NIAID, Rocky Mountain Laboratories, Hamilton, MT). The Huh 7 cells used by the CDC were obtained from Apath, LLC (Brooklyn, NY, USA). All cells were maintained following recommended protocols. Fresh human MDMs were generated and characterized at the Integrated Research Facility (IRF) immunology core laboratories [17].

### Virus Isolation

All procedures using infectious EBOV were performed under biosafety level 4 (BSL-4) conditions at the IRF or the CDC. An isolate of the EBOV Kikwit variant was obtained by the CDC from a patient specimen in 1995 (full designation: Ebola virus/H.sapiens-tc/COD/1995/Kikwit-9510621, abbreviation: EBOV/Kik). Following two passages of the virus in Vero E6 cells and an additional two passages in Vero E6 cells at the US Army Medical Research Institute of Infectious Diseases (USAMRIID), stocks for use in these studies were propagated at the IRF in BEI NR-596 Vero E6 cells and used after one or two passages (p1 or p2). The C05 isolate of the Makona variant of EBOV (full designation: Ebola virus/H.sapiens-tc/GIN/2014/Makona-C05, abbreviation: EBOV/Mak) was isolated in 2014 in Vero E6 cells and kindly provided by Dr. Gary P. Kobinger (Public Health Agency of Canada, Winnipeg, CA). For these studies, the virus was passaged two additional times in BEI NR-596 Vero E6 cells. A recombinant EBOV (Mayinga variant, Democratic Republic of Congo) encoding the enhanced green fluorescent protein (EBOV-eGFP) gene was generated by inserting the reporter eGFP gene between NP and VP35 as described previously [18].

The Hartley guinea pig-adapted EBOV (full designation: Ebola virus/UTMB/C.porcellus-lab/COD/1976/Mayinga-GPA, abbreviation: GPA-EBOV/May; BioSample SAMN05755733, GenBank accession no. TBD) was propagated in Vero cells and guinea pigs with the following sequence (Vero p1, GP p4, Vero p1, GP p3, Vero p1). This adapted virus was obtained from Dr. Thomas Geisbert (University of Texas Medical Branch at Galveston, Galveston, TX, USA) and passaged two additional times in BEI NR-596 Vero E6 cells prior to use in these studies. To generate virus working stocks, Vero E6 cells were infected at a multiplicity of infection (MOI) of 0.01. Following 5 to 7 days (d) of incubation, when cytopathic effects were visible, cell culture supernatants were collected and clarified by centrifugation. All EBOV isolates were titrated by plaque assay using Vero E6 cells.

### Human Primary MDM Generation and Characterization

Fresh human whole blood was obtained from the National Cancer Institute (NCI)-Frederick, MD, Research Donor Program, NIH blood bank, (Bethesda, MD, USA), Biological Specialty Corporation (Colmar, PA, USA) and Bioreclamation IVT (Long Island, NY, USA). The method for generating MDMs was described previously [17]. In brief, peripheral blood mononuclear cells (PBMCs) were isolated by density-gradient centrifugation over Histopaque (1.077 g/ml, Sigma-Aldrich). CD14^+^ cells were isolated using the human CD14 enrichment kit following the manufacturer’s instructions (Miltenyi Biotec, San Diego, CA). CD14^+^ monocytes were differentiated into MDMs by addition of 5 ng/ml of recombinant human macrophage colony-stimulating factor (rhM-CSF, Life Technologies, Grand Island, NY) and conditioned medium from KPB-M15 cells (kind gift from Dr. Atsunobu Hiraoka, SCGF Research Laboratory, Kyoto, JP) followed by 6 to 7 d of incubation at 37°C and 5% CO_2_. Medium was replaced every 2 to 3 d. Cells were treated with enzyme-free cell dissociation buffer (GIBCO Invitrogen, Carlsbad, CA). Both adherent and non-adherent cells were harvested and seeded at 1 x 10^5^ cells/well in black opaque or clear bottom 96-well Operetta plates (PerkinElmer, Waltham, MA) and then incubated at 37°C and 5% CO_2_ overnight for drug evaluation.

The harvested MDMs saved for immunophenotyping were washed with wash buffer (BD Biosciences, San Jose, CA, USA) and pelleted by centrifugation. Cell pellets were resuspended in 100 μl of a premixed macrophage characterization antibody panel, containing anti-human monoclonal antibodies specific for CD14-FITC, HLA-DR-V500, CD11b-Pacific blue™, CD163-APC, CD86-PE-Cy7 (BD Biosciences) and LIVE/DEAD^®^ Fixable Yellow Dead Cell Stain (Life Technologies, Grand Island, NY) along with appropriate isotype control (irrelevant mouse or hamster IgG1, IgG2a or IgG2b) antibody directly conjugated to fluorochrome. Cells were incubated in the dark at room temperature for 30 minutes (min). Cells were then washed once, and the pellets were resuspended in 300 μl of staining buffer (BD Biosciences). The stained MDMs were acquired on a BD Fortessa LSR II flow cytometer (BD Biosciences, San Jose, CA, USA). Results were analyzed using Flowjo software (FLOWJO, Ashland, OR), and cells were gated around the live, singlet monocyte population, which was strongly positive for CD11b and HLA-DR. Additional analysis of cell surface marker expression was based on the double-positive CD11b and HLA-DR population [17].

### Cell-based Efficacy and Cytotoxicity Testing of EBOV Antiviral Agents

Vero E6, HeLa, Huh 7, or Hep G2 cells were seeded at 3–4 x 10^4^ cells/well, and MDMs were plated at 1 x 10^5^ cells/well in black opaque or clear bottom 96-well Operetta plates and incubated at 37°C and 5% CO_2_ overnight. Drugs not already in liquid form were dissolved in dimethyl sulfoxide (DMSO) (Sigma-Aldrich). Final DMSO concentrations did not exceed 0.05%. After 24 hours (h), drug solutions were diluted in Dulbecco’s modified Eagles’s medium (DMEM) with 10% fetal bovine serum (FBS) to a 4X concentration in a 96-well drug dilution plate. Drugs were transferred from the drug dilution plates to cell plates (50 μl/well) using the 96-well liquidator (Rainin Instrument, Oakland, CA) at the time point specific for each experiment.

Two cell plates for each cell type were infected with EBOV and used for detecting viral inhibition. Cells were infected at specified MOIs by adding virus diluted in DMEM with 10% FBS directly to the drug mixture at the specified time point (final volume of 200 μl). After 48 or 72 h, cells were fixed with 10% neutral-buffered formalin. EBOV was detected by exposure to either a mouse antibody against the EBOV VP40 matrix protein (# B-MD04-BD07-AE11, made by USAMRIID under CDC contract) [19] or a human antibody against the EBOV glycoprotein (KZ52-IgG1, kindly provided by Dr. Dennis R. Burton, The Scripps Research Institute, La Jolla, CA) for 1–2 h at 37°C. Cells were stained with either Alexa Fluor^®^ 594 goat anti-mouse IgG (heavy + light chains) antibody (Life Technologies), anti-mouse IgG-peroxidase labeled antibody (Kirkegaard & Perry Laboratories [KPL], Gaithersburg, MD), or anti-human IgG, peroxidase labeled antibody (KPL). Fluorescence was quantified by a plate reader (Infinite^®^ M200, Tecan US, Morrisville, NC) or by an Operetta high-content imager (Perkin-Elmer, Waltham, MA). Luminescence was quantified using the SuperSignal enzyme-linked immunosorbent assay (ELISA) Pico Chemiluminescent Substrate (Thermo Fisher Scientific Inc., Rockford, IL) and the same plate reader (Tecan).

For all experiments, parallel cytotoxicity assays were performed to measure drug toxicity. One black opaque cell plate for each cell type was mock infected using DMEM + 10% FBS (no virus) under the same conditions as the infected cells, and cell viability was measured using the CellTiter Glo Luminescent Cell Viability Assay kit (Promega, Madison, WI) according to the manufacturer’s instructions. Luminescence was read on the Infinite^®^ M1000 Pro plate reader (Tecan). For the high-content imaging on the Operetta, cytotoxicity of the drugs was measured by quantifying nuclei of infected cells that were stained with the Hoechst 33342 dye (Life Technologies). Assays with EBOV-eGFP were performed at CDC as described previously [20]. Briefly, Huh 7 cells were seeded at 1 x 10^4^ cells per well on a black opaque 96-well plate. The following day, compounds were serially diluted in DMSO and added to the cells to yield a final DMSO concentration of 0.5%. After 1 h, the cells were infected with EBOV-eGFP at an MOI of 0.2. At 48 h post-inoculation, eGFP fluorescence was measured using a Synergy H1MD plate reader (BioTek, Winooski, VT). Concurrently, cell viability was determined by CellTiter-Glo luminescent cell viability assay (Promega) with compound-treated, mock-infected cells, as indicated above.

### Testing Drug Combinations for Synergy

The National Center for Advancing Translational Sciences (NCATS/NIH) prepared drug 6 x 6 matrix plates by acoustically dispensing drugs using an acoustic transfer system-100 (EDC Biosystems, Freemont, CA). DMEM was added to drug plates, and plates were shipped frozen to the IRF. Drug plates were thawed overnight at 4°C, briefly centrifuged, and then mixed, and drugs were transferred to cell plates for efficacy and cell viability assays. The cells were infected at an MOI of 0.1 with EBOV/Mak, and the assay endpoint was 72 h.

### Evaluation of Lamivudine Efficacy in Guinea Pigs

Male and female Hartley guinea pigs (gender balanced; 11- to 13-weeks old) were obtained from Charles River Laboratories (Wilmington, MA). Animals were doubly housed in micro-isolator cages with CareFresh bedding, and provided Teklad Global High Fiber Guinea Pig Diet (#2041) and purified (reverse osmosis) water *ad libitum*. Two experimental groups (n = 6) were treated orally with pharmaceutical-grade lamivudine at 20 or 60 mg/kg once daily beginning 3 d before challenge through 9 d post-challenge. In parallel, animals in the control group (n = 7) were orally administered sterile water. All three groups of animals were challenged with a target dose of 1000 plaque forming units (pfu) (back titer was 1300 pfu) of EBOV/May-GPA via intraperitoneal (IP) delivery at d 0. Disease progression and weight were monitored by daily observation.

### Evaluation of the Pharmacokinetics of Lamivudine in Guinea Pigs

Male and female Hartley guinea pigs (gender balanced; 6–7 weeks old) were obtained from Charles River Laboratories (Stone Ridge, NY). Jugular vein catheters were placed by the vendor prior to shipment. Animals were singly housed in hanging polycarbonate solid-bottom microisolator cages with hardwood chip bedding, and provided Harlan Teklad Certified Guinea Pig Chow (#2040C) and purified (reverse osmosis) water *ad libitum*. Animals were administered lamivudine via oral gavage at a dose of 60 mg/kg and a volume of 6 ml/kg body weight. Blood (~300 μl; processed to ~120 μl of plasma) was collected from the jugular vein catheter port at 0.25, 0.5, 1, 2, 3, 4, 6, 8, 24, 48 and 72 h post-dose. Lamivudine plasma concentrations were determined by liquid chromatography with tandem mass spectrometry (LC-MS/MS). LC-MS/MS was performed using a QTrap 5500 (AB Sciex, Framingham, MA) in multiple reaction monitoring mode MRM) and an Eclipse Plus C18 (Agilent Technologies, Santa Clara, CA), 50 x 2.1 mm, 3.5 μm column, using gradient elution with 0.1% formic acid in water and 0.1% formic acid in acetonitrile as the mobile phase. Lamivudine was extracted from plasma samples using plasma protein precipitation with acetonitrile, and a stable isotope form of lamivudine (M+5) was used in the method as an internal standard. The lower limit of quantitation (LLOQ) of the method was 2 ng/ml. Pharmacokinetic parameters were analyzed using Phoenix^®^ WinNonlin^®^ (version 6.3) software (Certara, Princeton, NJ) to perform noncompartmental modeling.

### Data analysis

Non-linear regression analysis was performed to calculate IC_50_s (GraphPad Software, La Jolla CA) using the dose-response curves for the drugs. Error bars of dose-response curves represent the standard deviation of three replicates unless indicated otherwise. The data sets for the 2- or 3-drug combinations were analyzed by NCATS, NIH in-house software using the Bliss interaction model [21, 22]. The ΔBliss metric described by Cokol et al. [23] was computed to determine synergy (ΔBliss <0), additive effects (ΔBliss >0), or non-interaction (ΔBliss = 0).

### Ethics Statement

Experimental infection of guinea pigs with EBOV was conducted in accordance with an Animal Study Protocol approved by the NIAID, Division of Clinical Research, Animal Care and Use Committee (Protocol #IRF-031E). Assessment of pharmacokinetics in guinea pigs was conducted in accordance with an Animal Study Protocol approved by the SRI International Animal Care and Use Committee. All animal studies were in compliance with the Animal Welfare Act regulations and the *Guide for the Care and Use of Laboratory Animals* recommendations. These institutions also accept as mandatory the Public Health Service policy on Humane Care of Vertebrate Animals used in testing, research and training. All animal work at NIAID and SRI International was performed in facilities accredited by the American Association for the Accreditation of Laboratory Animal Care International. All inoculations and treatments were performed under isoflurane anesthesia, and all efforts were made to minimize suffering. Disease progression and weight were monitored by daily observation. Animals were euthanized at the presence of one or more clinical signs of severe pain or distress (i.e., scruffy appearance; unresponsive, hunched; unable to move, comatose; agonal breathing; paralysis; head tilt with circling/rolling; unable to eat/drink; persistent scratching; eyes closed or slit; tremors). Euthanasia by inhalation of CO_2_ was confirmed by generation of a pneumothorax. No unexpected deaths occurred in this study.

## Results

### Effect of Lamivudine and Zidovudine on EBOV Replication

**Fig 1:**
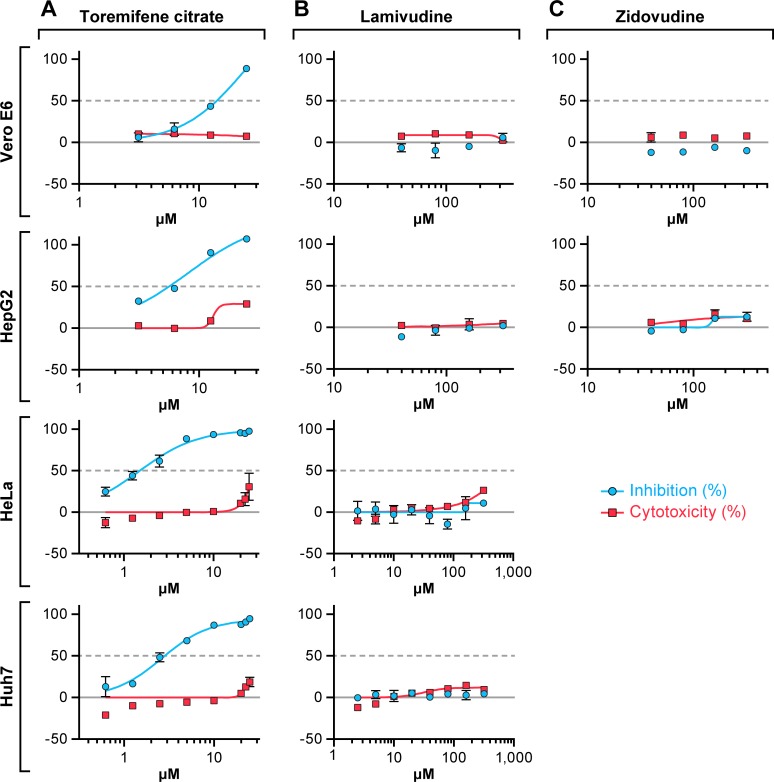
Antiviral activity against Kikwit variant of Ebola virus. Vero E6, Hep G2, HeLa, or Huh 7 cells were treated 1 h with toremifene citrate (A), lamivudine (B), or zidovudine (C). Two-fold dilutions of the drugs were tested in a 4- to 8-point dose-response curve. Then cells were infected at a multiplicity of infection (MOI) of 1 for 48 h. Toremifene citrate was used as a positive control. Antiviral activity is shown in blue and cytotoxicity is shown in red. The experiment was run on duplicate plates with triplicate wells per dose (mean ± SD; n = 3). Representative graphs from 1 to 4 independent experiments are shown.

The effect of four or eight point 2-fold dilutions of lamivudine and zidovudine on EBOV replication was evaluated using a cell-based ELISA in the established cell lines Vero E6, HeLa, Huh 7, and Hep G2. Toremifene citrate, an estrogen receptor antagonist that inhibits EBOV both *in vitro* and *in vivo* [24], was used as a positive control. Lamivudine and zidovudine had no detectable antiviral activity against EBOV/Kik in any cell line infected at an MOI of 1. Both drugs demonstrated minimal cytotoxicity in all cells lines examined (Fig 1). In contrast, toremifene citrate inhibited EBOV/Kik effectively in Vero E6, Hep G2, HeLa, and Huh 7 cells with IC_50_s ranging from 2.2–9.9 μM (Table 1).

**Table 1:** Effects of Lamivudine, Zidovudine, and Toremifene Citrate on Ebola Virus Replication.

					IC_50_ (μM)^d^		
Virus (variant)	MOI	Pre-treatment (h)	Assay (hpi)	Cell type	Lamivudine	Zidovudine	Toremifene
EBOV (Kik)	1	1	48	Vero E6	>320	>320	9.9 ± 4.4
EBOV (Kik)	1	1	48	Hep G2	>320	>320	3.5 ± 2.0
EBOV (Kik)	1	1	48	HeLa	>320	nd	2.2 ± 0.7
EBOV (Kik)	1	1	48	Huh 7	>320	nd	3.4 ± 0.4
EBOV (Kik)^a^	1	1	48	MDM (type 1)	>320	>320	~ 25
EBOV (Kik)^a^	1	1	48	MDM (type 2)	>320	>320	18.3 ± 0.1
EBOV (Mak)	1	1	48	Vero E6	>320	>320	7.2 ± 1.6
EBOV (Mak)	1	1	48	HeLa	>320	nd	1.41 ± 0.1
EBOV (Mak)	1	1	48	Huh 7	>320	nd	1.2 ± 0.2
EBOV (Mak)	0.4	1	48	Vero E6	>320	>320	2.4 ± 1.1
EBOV (Mak)	0.2	1	48	Huh 7	>320	>320	1 ± 0.1
EBOV (Mak)	0.1	1	72	Vero E6	>320	>320	1.7 ± 1.2
EBOV (Mak)	0.1	1	72	Huh 7	>320	>320	1.2 ± 0.1
EBOV (Mak)	0.1	1	72	MDM (type 1)	>320	>320	4.5 ± 0.9
EBOV (Mak)	0.1	24	48	Vero E6	>320	>320	3.5 ± 0.9
EBOV (Mak)	0.1	24	48	Huh 7	>320	35.4 ± 13	0.7 ± 0.2
EBOV (Mak)	0.1	24	48	MDM (type 1)	>320	>320	2.1 ± 0.3
EBOV (GPA)	0.1	1	48	Huh 7	>320	**-**	1.8 ± 0.5
HIV-1				Macrophages	0.002^b^	**-**	**-**
HIV-1				Monocytes	0.69^b^	**-**	**-**
HIV-1				PBMC	0.002–2.5^b^	**-**	**-**
HBV				Hep G2 (2.2.15)	0.002^c^	**-**	**-**

EBOV, Ebola virus; GPA, guinea pig adapted; HBV, hepatitis B virus; HIV-1, human immunodeficiency virus-1; hpi: hours post-inoculation; IC_50_, 50% inhibitory concentration; Kik, Kikwit; Mak, Makona; MDM, monocyte-derived macrophage; MOI, multiplicity of infection; nd, not determined; PBMC, peripheral blood mononuclear cell.

^a^Data from Hensley at al. (2015) [15]

^b^Data from Schinazi et al. (2003) [25].

^c^Data from Kruining et al. (1995) [14].

^d^ IC_50_ values are mean values ± the standard deviation (SD) from 2–6 dose response curves.

**Fig 2:**
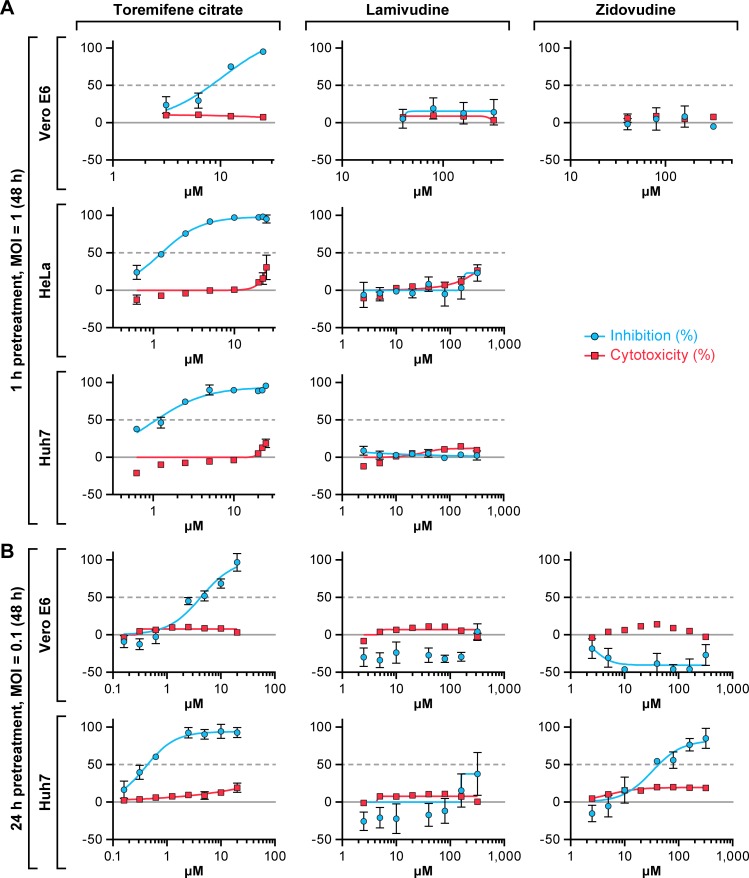
Antiviral activity against Makona variant of Ebola virus at an MOI of 1 or 0.1. (A) Vero E6, HeLa, or Huh 7 cells were treated with toremifene citrate, lamivudine, or zidovudine for 1 h. Then cells were infected with EBOV/Mak at a multiplicity of infection (MOI) of 1 for 48 h. (B) Vero E6 or Huh 7 cells were treated with toremifene citrate, lamivudine, or zidovudine for 24 h. Then cells were infected with EBOV/Mak at a multiplicity of infection (MOI) of 0.1 for 72 h. Toremifene citrate was used as a positive control. Antiviral activity is shown in blue and cytotoxicity is shown in red. The experiment was run on duplicate plates with triplicate wells per dose (mean ± SD; n = 3). Representative graphs from 1 to 6 independent experiments are shown.

Lamivudine and zidovudine were also tested for activity against the variant EBOV/Mak. These HIV-1 drugs did not show antiviral activity when assayed at 48 h post-inoculation in Vero E6, Hela, or Huh 7cells infected at an MOI of 1 (Fig 2A). Lowering the MOI to 0.1 had no impact on the antiviral activity of either drug with 1 h pre-treatment and 72 h as an endpoint (Table 1). When extending drug pre-treatment to 24 h at an MOI of 0.1, low EBOV inhibition was only observed in Huh 7 cells with zidovudine at the two highest concentrations tested with an IC_50_ of 35.4 μM (Fig 2B). No significant anti-EBOV activity was observed for lamivudine. Over a series of six separate experiments, minimal effects at the highest concentration tested (320 μM) were noted, however the effects were insufficient to calculate an IC_50_. In contrast to lamivudine, toremifene citrate continuously showed strong antiviral activity against EBOV in all cell lines under each condition (Fig 2A and 2B, Table 1). The effect of extended drug pre-treatment was further evaluated in Vero E6 and Huh 7 cells. The antiviral activity did not improve when cells were treated with lamivudine for up to 48 h prior to infection with EBOV/Mak (S1 Fig).

A recent publication reported that HIV-1 drugs inhibit EBOV virus-like particles (trVLPs) that undergo a single cycle of transcription and replication [16]. The drugs showed activity alone or in combination against the transcription-competent trVLPs, but limited efficacy against EBOV-eGFP [16]. To investigate further these findings, we repeated the studies using the same cell lines, compound source, time of drug addition, and length of infection time to compare with the conditions we used to perform the drug screen study on EBOV/Mak (S2 Fig). In 293T, Vero E6, and Huh 7 cells, lamivudine from two vendors (ViiV Healthcare; Sigma Aldrich) did not demonstrate any antiviral activity regardless of the treatment protocol (Table 2, S2 Fig). The positive control, toremifene citrate continued to demonstrate strong antiviral activity against EBOV/Mak in all cell lines under each condition (Table 2).

**Table 2:** Effects of Lamivudine from Different Sources on Ebola Virus Replication.

Virus (variant)	MOI	Pre-treatment(h)	Post-treatment (h)	Assay (hpi)	Cell type	IC_50_ (μM)^a^		
						Lamivudine (ViiV Healthcare)	Lamivudine (Sigma)	Toremifene
EBOV/Mak	0.1	1	-	72	Vero E6	>320	>320	1.58 ± 0.1
EBOV/Mak	0.1	1	-	72	Huh 7	>320	>320	0.89 ± 0.3
EBOV/Mak	0.1	1	-	72	293T	>320	>320	0.56 ± 0.1
EBOV/Mak	0.1	-	24	72	Vero E6	>320	>320	7.21 ± 1.5
EBOV/Mak	0.1	-	24	72	Huh 7	>320	>320	2.14 ± 0.5
EBOV/Mak	0.1	-	24	72	293T	>320	>320	2.97 ± 0.3

EBOV, Ebola virus; hpi, hours post-inoculation; IC_50_: 50% inhibitory concentration; Mak, Makona; MOI, multiplicity of infection.

^a^ IC_50_ values are mean values ± the standard deviation from 4 dose-response curves.

### Effect of Lamivudine and Zidovudine on EBOV Replication in Human MDMs

**Fig 3:**
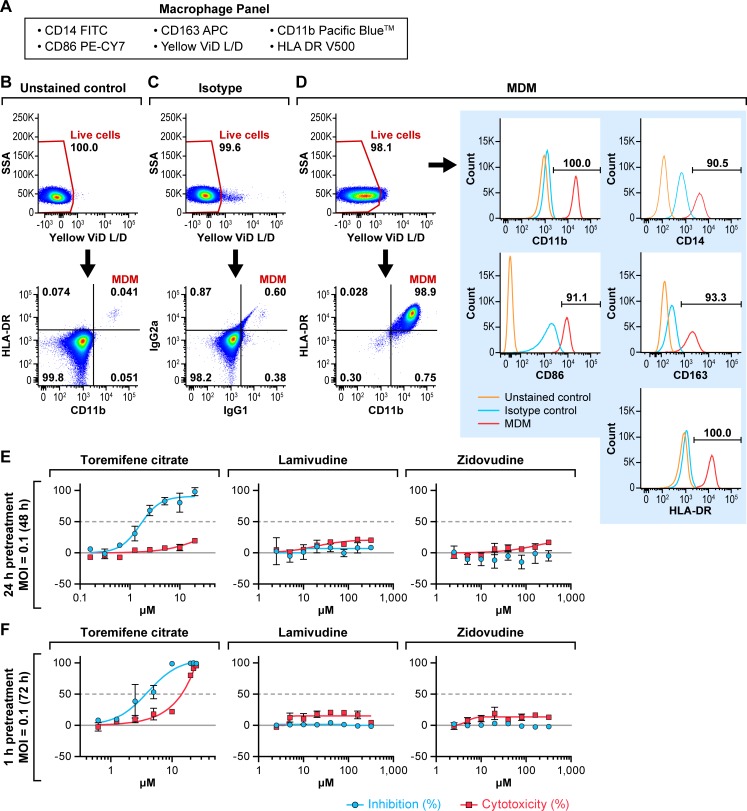
Antiviral activity of lamivudine and zidovudine against EBOV/Mak in MDMs. (A) MDMs were surface stained with the macrophage panel markers listed. (B) The unstained control sample was stained with LIVE/DEAD Fixable Yellow Dead Cell Stain (Yellow ViD L/D) only. (C, D) The MDM population was measured according to double-positive CD11b and HLA-DR gating. The expression of individual macrophage markers (red line) were gated based on the isotype control (blue line). The percentage of cells positive for each marker is indicated (> 90%). (E, F) MDMs were treated with toremifene citrate, lamivudine, or zidovudine and infected with EBOV/Mak at a multiplicity of infection (MOI) of 0.1. (E) MDMs were pre-treated for 24 h before infection, and the assay endpoint was 48 h post-inoculation. (F) MDMs were pretreated for 1 h before inoculation, and the assay endpoint was 72 h post-inoculation. Toremifene citrate was used as a positive control. Antiviral activity is shown in blue and cytotoxicity is shown in red. The experiment was run on duplicate plates with triplicate wells per dose (mean ± SD; n = 3). Representative graphs from 1 to 4 independent experiments are shown.

Macrophages are thought to be among the earliest cells targeted by EBOV *in vivo* and play a role in facilitating viral dissemination following infection [26–31]. Newly differentiated MDMs were characterized by flow cytometry by analyzing the expression of major macrophage markers after surface staining, including HLA-DR, CD11b, CD14, CD163, and CD86, to confirm that the MDM population was mature and highly purified (Fig 3A–3D). The isolated MDMs were predominantly CD14^+^, CD11b^+^, HLA-DR^+^, CD163^+^, and CD86^+^, and over 98% of the cells were positive as macrophages compared to that observed with the isotype control (Fig 3D). Overall, MDMs retained viability throughout manipulation (98.5 ± 1.1%). The highly purified MDMs were pretreated for 24 h or 1 h with the HIV-1 drugs at doses ranging from 0–320 μM and infected with EBOV/Mak at an MOI of 0.1 for 48 h or 72 h, respectively (Fig 3E and 3F). Toremifene citrate inhibited EBOV replication with IC_50_s ranging from 2.1 to 4.5 μM (Table 1), and cytotoxicity increased at doses of 10 μM and above in the 72-h assay (Fig 3F). In contrast, lamivudine and zidovudine did not demonstrate any antiviral activity against EBOV/Mak infection (Fig 3E and 3F).

### Effect of Lamivudine and Zidovudine on EBOV-eGFP Replication

**Fig 4:**
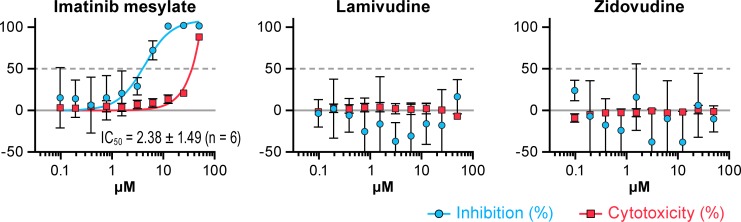
Antiviral activity of lamivudine and zidovudine against EBOV-eGFP virus. Huh 7 cells were pre-treated with imatinib mesylate, lamivudine or zidovudine for 1 h and then inoculated with EBOV-eGFP virus at a multiplicity of infection (MOI) of 0.2. Fluorescence was determined at 48 h post-inoculation. Imatinib mesylate was used as a positive control. Antiviral activity is shown in blue and cytotoxicity is shown in red. The experiment was run with a ten-point, two-fold dilution scheme with quadruplicate wells per dose.

To characterize further the effect of certain HIV-1 drugs on EBOV replication, lamivudine and zidovudine were evaluated at the CDC for their ability to inhibit the replication of EBOV-eGFP. None of these compounds showed any anti-EBOV activity except for imatinib mesylate [32] that was used as a positive control (Fig 4).

Data obtained in this study for lamivudine and zidovudine are summarized in Tables 1 and 2. Historical data on the antiviral effect of lamivudine against HIV-1 and HBV were included for comparison [14, 24, 25]. Lamivudine has been reported to have high potency against HIV-1 and HBV in Hep G2 cells and primary cells (e.g., PBMCs, macrophages). The drug had a complete lack of activity against the two isolates of EBOV even at the highest doses tested (up to 320 μM).

### Effect of Combinations of HIV-1 Drugs on EBOV Replication

**Fig 5:**
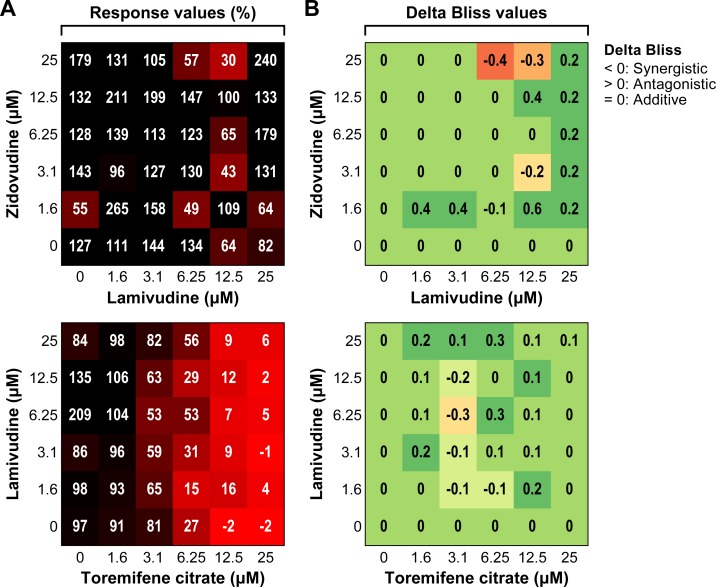
Antiviral activity of drug combinations against Makona variant of Ebola virus. Dose matrix (6 x 6) evaluation of two drug combinations, lamivudine/toremifene citrate and lamivudine/zidovudine. Huh 7 cells were pre-treated with drug combinations for 1 h and inoculated at a multiplicity of infection (MOI) of 0.1 for 72 h. (A) The heat map of percent response shows the antiviral activity of each combination (100% corresponds to “no activity”). (B) The ΔBliss plot indicates how much a combination effect differs from the additive effect as determined by the Bliss model. The experiment was run once with triplicate wells per dose (mean ± SD; n = 3). ΔBliss = 0 (additive effect); ΔBliss <0 (synergistic effect); ΔBliss >0 (antagonistic effect).

To investigate the possibility of a synergistic effect, dual and triple combination studies were performed using toremifene citrate and HIV-1 drugs, lamivudine and zidovudine. None of the combinations showed an appreciable synergistic effect. The heat maps for percent response and the ΔBliss plots are shown for two combinations (Fig 5). The ΔBliss values for lamivudine/toremifene citrate or lamivudine/zidovudine combinations indicate that no appreciable synergistic interaction was observed with either combination. The activity observed for the lamivudine/toremifene citrate combination is due to toremifene citrate alone (Fig 5A).

### Evaluation of Pharmacokinetics of Lamivudine in Guinea Pigs

**Fig 6:**
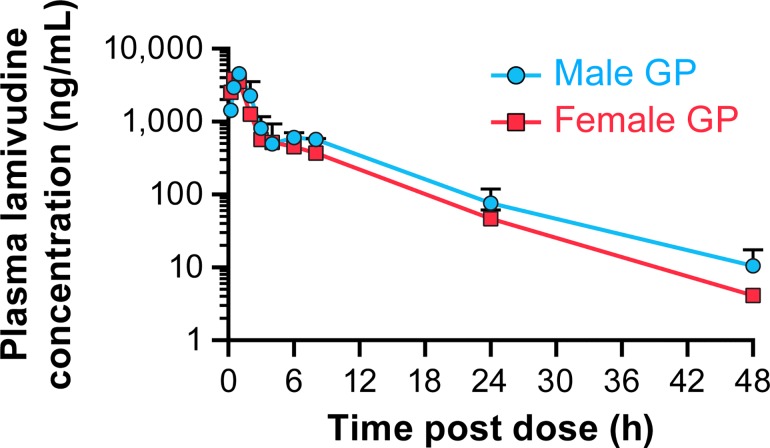
Pharmacokinetic study of lamivudine in guinea pigs. Plasma concentrations of lamivudine in male and female Hartley guinea pigs after a single oral dose, 60 mg/kg. Each data point represents the mean ± SD of n = 3 guinea pigs except for the 48 h time point in females, n = 2.

Efficacy of lamivudine was evaluated in the guinea pig model for EVD. To ensure that plasma levels of lamivudine in guinea pigs could be achieved that are equivalent to plasma levels in humans being treated for AIDS, pharmacokinetics of the drug in guinea pigs were evaluated. The study was performed with the maximum dose of 60 mg/kg (3 times the equivalent dose of 300 mg in humans). All animals were administered lamivudine via oral gavage at a dose of 60 mg/kg and showed no adverse clinical signs and appeared normal throughout the study. Lamivudine was readily absorbed and reached peak plasma concentrations by 1 h (Fig 6). The plasma concentrations exhibited a slight biphasic profile with a small second peak at approximately 4–6 h. Plasma concentrations of lamivudine were below the lower limit of quantitation (LLOQ = 2 ng/ml) in all animals by 72 h. Plasma concentrations of lamivudine tended to be higher in males compared with females (S1 Table). Lamivudine reached peak plasma concentrations at T_max_ at 0.5 or 1 h post-dose. The maximum plasma drug concentration (C_max)_ was 4560 ng/ml (males) and 4107 ng/ml (females). The area under the concentration-time curve extrapolated to infinity (AUC_inf_) was about 34% higher in male guinea pigs compared with the females, 16837 h·ng/ml versus 12584 h·ng/ml. The elimination half-life (t_1/2_) for the terminal phase of lamivudine varied from 5.4 h (Female #5) to 8.3 h (Male #1). The apparent total clearance of the drug from plasma (Cl/F) was 3575 ml/h/kg (males) and 4908 ml/h/kg (females). The apparent volume of distribution (Vz/F) was very high, ~35 l/kg and 45 l/kg in male and female guinea pigs, respectively, indicating probable intracellular distribution.

### Evaluation of efficacy of lamivudine in a guinea pig model of EVD

**Fig 7:**
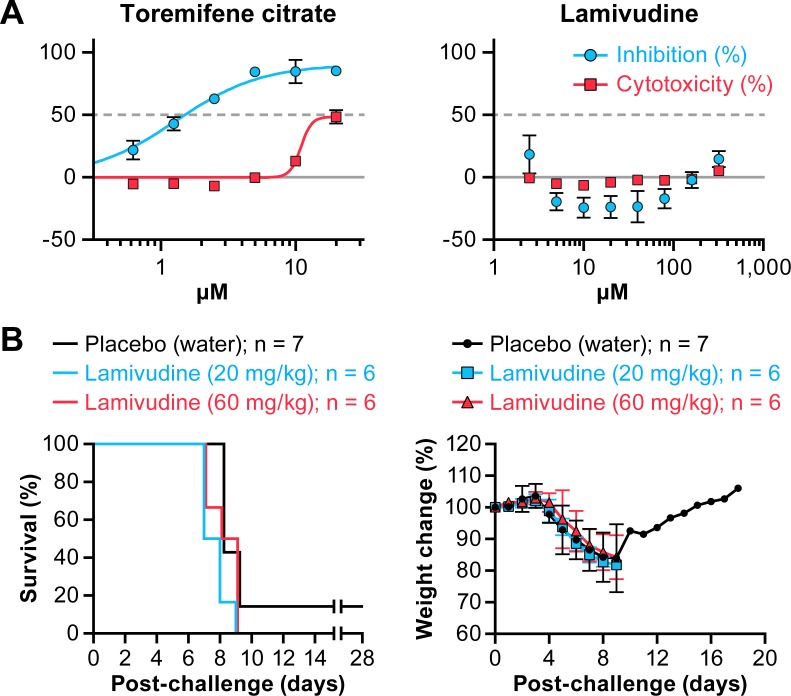
***In vitro* and *in vivo* evaluation of lamivudine activity against GPA- EBOV** (A) Huh 7 cells were pre-treated with toremifene citrate or lamivudine for 1 h. Cells were then inoculated at a multiplicity of infection (MOI) of 0.1 for 48 h. Antiviral activity is shown in blue and cytotoxicity is shown in red. The experiment was run on duplicate plates with triplicate wells per dose (mean ± SD; n = 3). Representative graphs from 2 independent experiments are shown. (B) Percent survival and weight loss of lamivudine-treated guinea pigs infected with guinea pig-adapted-Ebola virus/Mayinga variant (GPA-EBOV/May). Two groups of guinea pigs (n = 6) received oral lamivudine at 20 mg/kg or 60 mg/kg daily from d 3 pre-exposure to d 9 post-exposure. In parallel, the mock group (n = 7) was treated with water. All animals were challenged intraperitoneally (IP) with approximately 1300 pfu of GPA-EBOV/May on d 0.

The effect of lamivudine on GPA-EBOV/May replication *in vitro* was evaluated in Huh 7 cells using cell-based ELISA. With 1 h pre-treatment and a 48 h assay endpoint, lamivudine showed no antiviral activity against GPA-EBOV/May in cells infected at an MOI of 0.1, while toremifene citrate inhibited GPA-EBOV/May effectively with an IC_50_ of 1.8 μM. (Fig 7A, Table 1).

Efficacy of lamivudine oral solution was then evaluated in the guinea pig model for EVD. Animals were orally treated once daily with 20 mg/kg (n = 6), the calculated equivalent to a 300 mg dose in humans or three times that dose (60 mg/kg; n = 6) of lamivudine, or water (n = 7) as mock controls. Oral treatment was initiated on d 3 before challenge and continued through d 9 post-challenge. Animals were challenged via the intraperitoneal (IP) route with 1300 pfu GPA-EBOV/May. All animals in the lamivudine treatment groups developed clinical signs of disease and succumbed by d 9 post-challenge (Fig 7B). One of seven animals in the mock-treatment group survived (14% survival), but developed clinical signs of disease (e.g., weight loss) and recovered without overt sequelae. These data demonstrate that lamivudine was not protective against EBOV infection in a guinea pig model of EVD.

## Discussion

Due to the lack of available drugs combined with the enormity of the EVD epidemic, researchers evaluated previously approved drugs that could be repurposed for treating patients with EVD. On the surface, the HIV-1 drugs, lamivudine and zidovudine, are attractive candidates since they are inexpensive and available in Western Africa. However, examination of the mechanism of action of lamivudine and zidovudine is a necessary scientific question to ascertain how these drugs could be efficacious against EBOV.

Lamivudine and zidovudine are synthetic nucleoside analog inhibitors of the reverse transcriptase enzyme of HIV-1 and HBV, *in vitro* and *in vivo* [12, 14, 25, 33]. Intracellularly, lamivudine and zidovudine are metabolized to the active triphosphate form by multiple kinases. The triphosphate form binds competitively to reverse transcriptase, and the incorporation of the triphosphate form into viral DNA results in chain termination [12]. However, EBOV is a negative-sense RNA virus that lacks reverse transcriptase and requires an RNA-dependent RNA polymerase. Therefore, if these drugs are active against EBOV, a mechanism other than inhibition of a reverse transcriptase would be operating.

Our laboratory previously assessed the efficacy of several HIV-1 inhibitors, including lamivudine, against EBOV/Kik in a small *in vitro* study, and we observed no discernable antiviral activity [15]. Additional studies were conducted to confirm these results and address additional speculations that lamivudine had measurable anti-EBOV activity *in vitro*. Specifically, experiments were performed using several cell lines and primary MDMs that serve as surrogates of target cells for EBOV infection *in vivo*. These experiments were designed to test multiple MOIs and pre- or post-treatment conditions. Despite all of the conditions tested, only a single experiment examining drug efficacy of lamivudine or zidovudine in one cell line (Huh 7) using a low MOI and 24-h drug pretreatment indicated that any anti-EBOV activity was observed. Lamivudine activity was minimal (less than 50% inhibition), zidouvudine activity was stronger (IC_50_ of 35.4 μM), but these activities were not consistently reproducible.

Recently, McCarthy et al reported that several HIV-1 drugs were potent at inhibiting replication of EBOV-trVLP, and these drugs also had limited efficacy against EBOV-eGFP [16]. McCarthy et al also identified drug combinations that had a synergistic effect for inhibiting EBOV replication. In our cell-based ELISA drug screen using the EBOV/Mak variant at a low MOI of 0.1, exposure of cells to the drug combinations of lamivudine, zidovudine, and toremifene citrate for 72 h did not demonstrate synergistic activity. McCarthy et al demonstrated limited antiviral activity for lamivudine against EBOV-eGFP with an IC_25_ = 43.13 μM without achieving greater than 45% inhibition of viral replication [16]. In addition to our work, concurrent and independent studies performed at the CDC evaluated the effect of the same anti-HIV-1 drugs on EBOV-eGFP infection in cell culture. These studies found no antiviral activity for lamivudine and zidovudine against EBOV infection, further confirming our *in vitro* data.

Despite lamivudine demonstrating minimal to no *in vitro* activity, there was some speculation that the drug could have off-target effects or survival benefit *in vivo* that would be missed by *in vitro* screens. To test this possibility, we performed an *in vivo* study to evaluate the efficacy of lamivudine in a guinea pig model of EBOV infection. We did not observe any protective activity of lamivudine in this model. While larger group sizes and additional models, doses, and regimens could be tested, the performance of these experiments cannot be supported with the data on hand.

The lack of *in vivo* activity of lamivudine could be attributed to poor bioavailability of the drug in guinea pigs; however, the C_max_ observed in guinea pigs was approximately 3 times higher than the C_max_ observed in humans from a 300 mg dose, with similar clearance and C_min_ levels [34]. These data confirm that the doses selected for the efficacy study in the guinea pig model were appropriate for comparison to human data.

The studies presented here do not support the hypothesis that lamivudine has a therapeutic effect in EVD patients. Supportive care may have played a role in the recovery of the 13 out of 15 EVD patients in Western Africa, although the confirmation of EVD on these patients was not mentioned. Unfortunately, documentation or detailed information regarding the study design, criteria for selection of the patients, and the disease development stage are not available.

During the EVD epidemic, the rate of new cases and the disease severity resulted in an urgent need to accelerate testing of experimental treatments as well as drugs approved for other indications such as lamivudine. While minimizing disease spread and severity in an outbreak setting is required, we consider it of equal importance to maintain the conventional practice of rigorous testing of potential drugs *in vitro* and/or in preclinical models before evaluation in EVD patients in the clinic.

## Supporting Information

S1 Fig Effect of pretreatment with lamivudine on the antiviral activity against Makona variant of Ebola virus. Vero E6 or Huh 7 cells were pretreated with lamivudine for 1, 24, or 48 h. Cells were then inoculated at a multiplicity of infection (MOI) of 0.1 for 72 h. Antiviral activity is shown in blue and cytotoxicity is shown in red. The experiment was run on duplicate plates with triplicate wells per dose (mean ± SD; n = 3). Representative graphs from 1 experiment are shown.
(TIF)

S2 Fig Antiviral activity of lamivudine from two vendors, Sigma and ViiV Healthcare. (A) Vero E6, Huh 7 or 293T cells were inoculated with EBOV/Mak at a multiplicity of infection (MOI) of 0.1 for 72 h. At 24 h post-inoculation, cells were treated with lamivudine from Sigma or ViiV Healthcare. Toremifene citrate was used as a positive control. Antiviral activity is shown in blue and cytotoxicity is shown in red. The experiment was run on duplicate plates with triplicate wells per dose (mean ± SD; n = 3). Representative graphs from 2 independent experiments are shown.
(TIF)

S1 Table Pharmacokinetic Parameters of Lamivudine in Male and Female Guinea Pigs after Oral Administration. (DOCX)
