# Supplementary figures and images for: Evaluation of the Activity of Lamivudine and Zidovudine against Ebola Virus
Source: PLoS One. 2016 Nov 30;11(11):e0166318. doi: 10.1371/journal.pone.0166318 (PMC5130197; doi:10.1371/journal.pone.0166318)

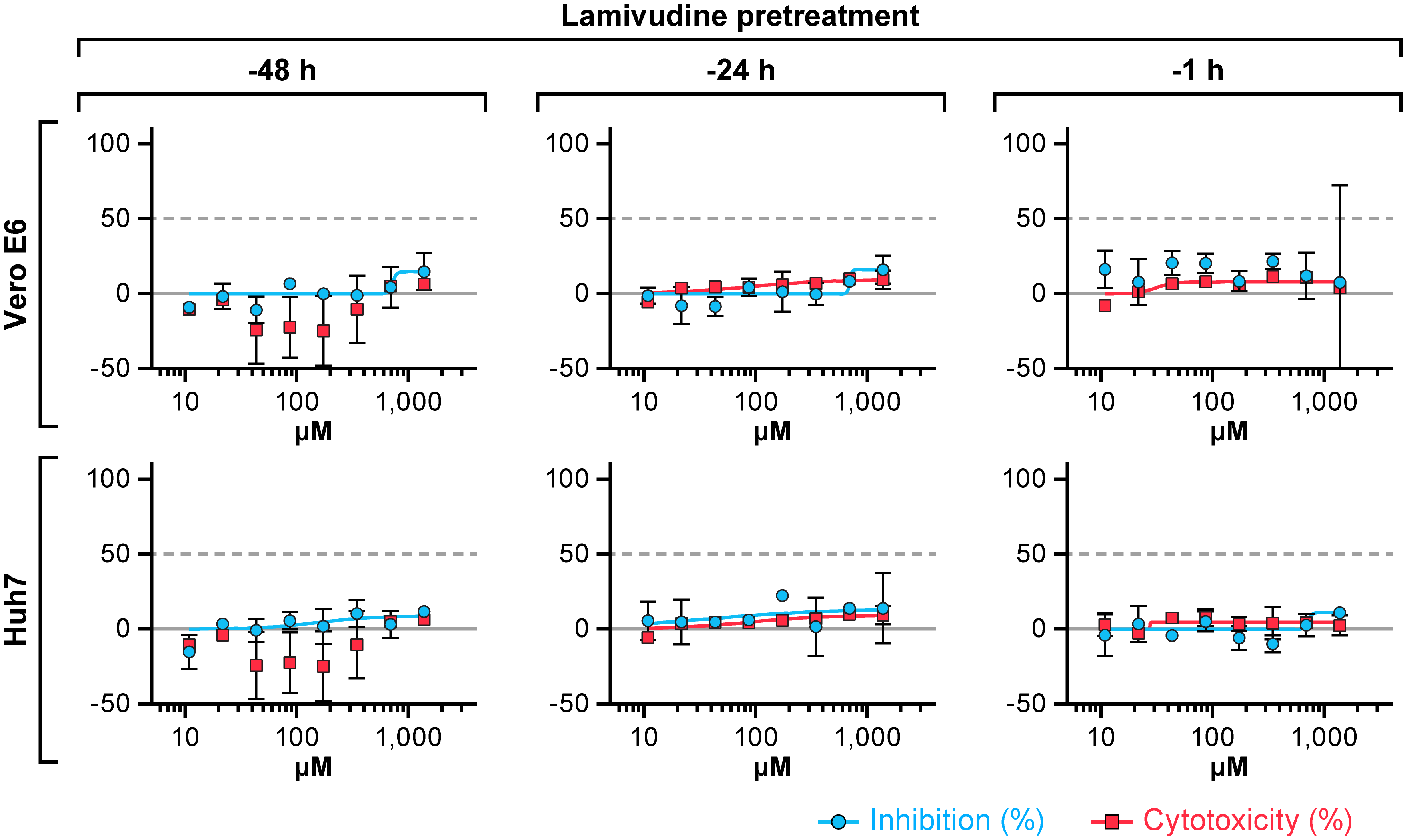

Supplement: S1 Fig — Vero E6 or Huh 7 cells were pretreated with lamivudine for 1, 24, or 48 h. Cells were then inoculated at a multiplicity of infection (MOI) of 0.1 for 72 h. Antiviral activity is shown in blue and cytotoxicity is shown in red. The experiment was run on duplicate plates with triplicate wells per dose (mean ± SD; n = 3). Representative graphs from 1 experiment are shown. (TIF) [file pone.0166318.s001.tif]

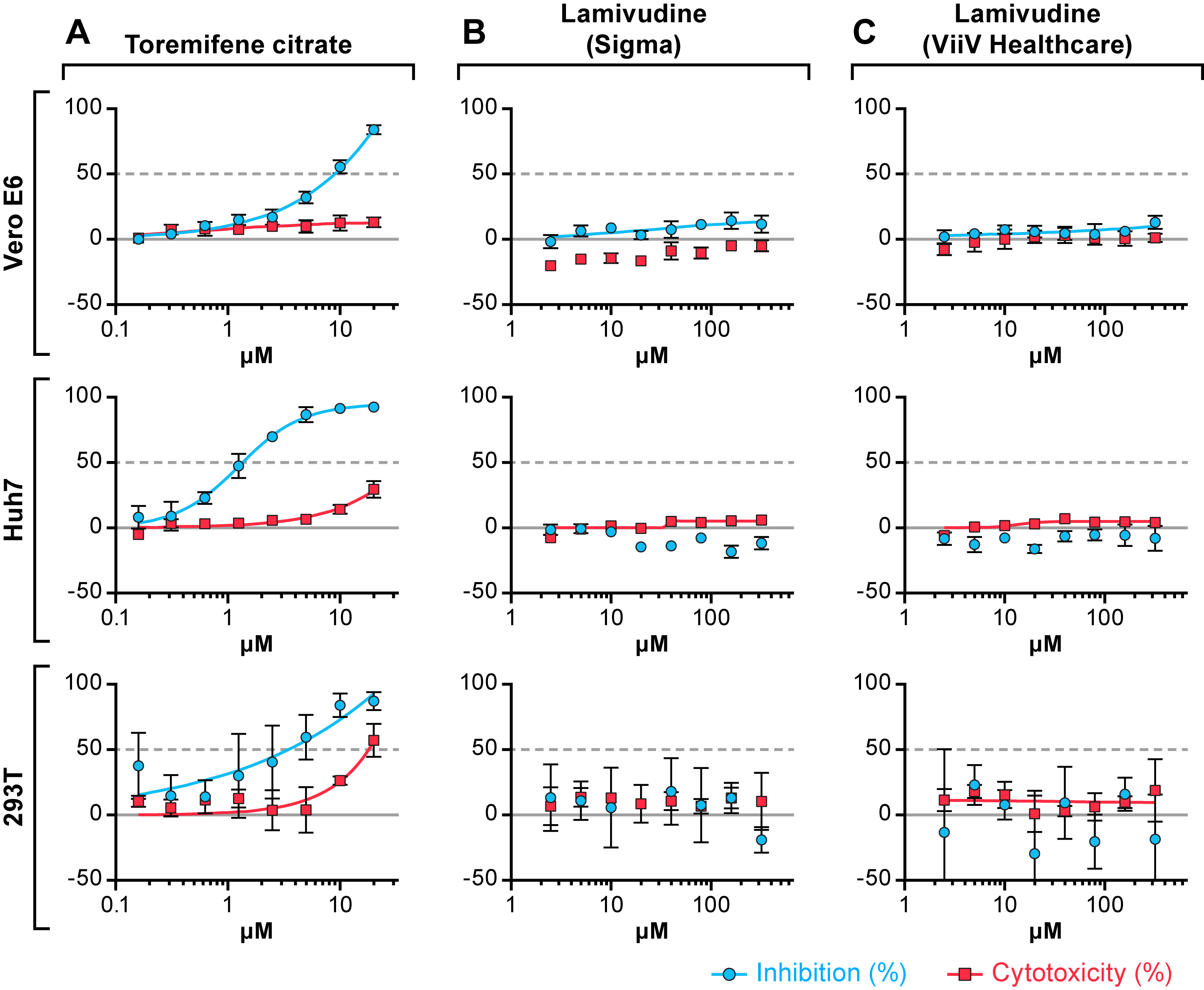

Supplement: S2 Fig — (A) Vero E6, Huh 7 or 293T cells were inoculated with EBOV/Mak at a multiplicity of infection (MOI) of 0.1 for 72 h. At 24 h post-inoculation, cells were treated with lamivudine from Sigma or ViiV Healthcare. Toremifene citrate was used as a positive control. Antiviral activity is shown in blue and cytotoxicity is shown in red. The experiment was run on duplicate plates with triplicate wells per dose (mean ± SD; n = 3). Representative graphs from 2 independent experiments are shown. (TIF) [file pone.0166318.s002.tif]
