# Supplementary material for: Evaluation of the Activity of Lamivudine and Zidovudine against Ebola Virus
Source: PLoS One. 2016 Nov 30;11(11):e0166318. doi: 10.1371/journal.pone.0166318 (PMC5130197; doi:10.1371/journal.pone.0166318)
Supplement: S1 Table — (DOCX) [file pone.0166318.s003.docx]

**S1 Table. Pharmacokinetic Parameters of Lamivudine in Male and Female Guinea Pigs after Oral Administration.**

|  |  |  | **C_max_ (ng/ml)** |  | **AUC_last_ (h·ng/ml)** | **AUC_inf_ (h·ng/ml)** | **Cl/F (ml/h/kg)** |  |
| --- | --- | --- | --- | --- | --- | --- | --- | --- |
| **Animal** | **Sex** | **T_max_ (hr)** |  | **t_1/2_(h)** |  |  |  | **Vz/F (ml/kg)** |
| 1 | M | 1.0 | 4270 | 8.3 | 15496 | 15718 | 3825 | 45797 |
| 2 | M | 1.0 | 5270 | *6.2* | 17276 | 17329 | 3465 | 30777 |
| 3 | M | 1.0 | *4140* | *6.0* | 17405 | 17464 | 3435 | 29698 |
|  | *Mean* | *1.0* | *4560* | *6.8* | *16726* | *16837* | *3575* | *35424* |
|  | *SD* | *0.0* | *618* | *1.3* | *1067* | *972* | *217* | *9000* |
| 4 | F | 0.5 | *4150* | 6.3 | 13320 | 13367 | 4489 | 40828 |
| 5 | F | 1.0 | 3860 | 5.4 | 14563 | 14586 | 4113 | 31783 |
| 6 | F | 1.0 | 4310 | 7.0 | 9497 | 9800 | 6123 | 61671 |
|  | *Mean* | *0.83* | *4107* | *6.2* | *12460* | *12584* | *4908* | *44761* |
|  | *SD* | *0.29* | *228* | *0.8* | *2640* | *2488* | *1068* | *15327* |

T_max_, peak plasma concentrations; C_max_, maximum plasma drug concentration; t_1/2_, elimination half-life; AUC_last_, area under the plasma concentration-time curve from time zero to time of last measurable concentration; AUC_inf_ , area under the concentration-time curve extrapolated to infinity; Cl/F, apparent total clearance of the drug from plasma; Vz/F, apparent volume of distribution; SD, standard deviation
